# Co-Infection with Three Mycoviruses Stimulates Growth of a *Monilinia fructicola* Isolate on Nutrient Medium, but Does Not Induce Hypervirulence in a Natural Host

**DOI:** 10.3390/v11010089

**Published:** 2019-01-21

**Authors:** Thao T. Tran, Hua Li, Duy Q. Nguyen, Michael G. K. Jones, Stephen J. Wylie

**Affiliations:** Plant Biotechnology Research Group-Virology, Western Australian State Agricultural Biotechnology Centre, School of Veterinary and Life Sciences, Murdoch University, Perth 6150, Australia; t.tran@murdoch.edu.au (T.T.T.); perthmuzi@yahoo.com (H.L.); q.nguyen@murdoch.edu.au (D.Q.N.); m.jones@murdoch.edu.au (M.G.K.J.)

**Keywords:** brown rot, stone fruit, *Prunus*, mycovirus, hypervirulence, hypovirulence, isogenic

## Abstract

*Monilinia fructicola* and *Monilinia laxa* are the most destructive fungal species infecting stone fruit (*Prunus* species). High-throughput cDNA sequencing of *M. laxa* and *M. fructicola* isolates collected from stone fruit orchards revealed that 14% of isolates were infected with one or more of three mycoviruses: Sclerotinia sclerotiorum hypovirus 2 (SsHV2, genus *Hypovirus*), Fusarium poae virus 1 (FPV1, genus *Betapartitivirus*), and Botrytis virus F (BVF, genus *Mycoflexivirus*). Isolate M196 of *M. fructicola* was co-infected with all three viruses, and this isolate was studied further. Several methods were applied to cure M196 of one or more mycoviruses. Of these treatments, hyphal tip culture either alone or in combination with antibiotic treatment generated isogenic lines free of one or more mycoviruses. When isogenic fungal lines were cultured on nutrient agar medium in vitro, the triple mycovirus-infected parent isolate M196 grew 10% faster than any of the virus-cured isogenic lines. BVF had a slight inhibitory effect on growth, and FPV1 did not influence growth. Surprisingly, after inoculation to fruits of sweet cherry, there were no significance differences in disease progression between isogenic lines, suggesting that these mycoviruses did not influence the virulence of *M. fructicola* on a natural host.

## 1. Introduction

Mycoviruses are viruses that infect fungi. They have been identified from all major fungal phyla, namely the Zygomycota, Chytridiomycota, Ascomycota, and Basidiomycota [1,2]. Since mycoviruses were first described [3], the partial or complete genomes of more than 250 mycoviruses have been sequenced [4]. Fungi can be multiply infected with closely-related and distantly-related viruses [5]. Most mycoviruses have double-stranded (ds) or single-stranded (ss) RNA genomes, and some groups do not encode a coat protein.

The influence that mycoviruses have on the ecology of their hosts is not well studied. Some mycoviruses reduce the ability of the fungal host to cause disease in plants. These are known as hypovirulent mycoviruses, and they have potential as biological control agents. The most well-known are Cryphonectria hypoviruses 1 and 2 (CHV1, CHV2), which significantly decreased the virulence of the fungus *Cryphonectria parasitica*, the causal agent of chestnut blight [6,7]. Other hypovirulent mycoviruses reduce the pathogenicity of white mold fungus *Sclerotinia sclerotiorum* [8,9] and white root rot fungus *Rosellinia necatrix* [10]. In contrast, several mycoviruses are associated with hypervirulence, described as a higher level of virulence or sporulation in their fungal hosts [11,12,13]. Other mycoviruses are reported to be associated with latent infections [8,14,15,16].

*Monilinia fructicola* and *M. laxa* were first recorded in Western Australian stone fruit production regions in 1997 [17,18], and they have since spread to all other stone fruit production regions in the state [19]. They incur costs in control (fungicides, gathering and destroying mummified fruit) and crop losses. Very little is known about mycoviruses that infect *Monilinia* species. Tsai, et al. [20] identified seven virus-like double-stranded RNA species in 36 of 49 *M. fructicola* isolates infecting nectarine and peach orchards in New Zealand. Although not characterized genetically, the authors described virus-like particles resembling those of partitiviruses, totiviruses, tobraviruses and furoviruses. They identified no differences in host growth rates between isolates with and without virus infection. In this current study, we identified three mycoviruses infecting a collection of *M. fructicola* and *M. laxa* isolates, and undertook to determine if these viruses influenced growth rates of infected fungal cultures in vitro, and the virulence of the pathogen on a natural host.

## 2. Materials and Methods

### 2.1. Fungus Collection and Isolation

Eighteen *M. laxa* and ten *M. fructicola* isolates were collected from symptomatic flowers, twig cankers and fruits from stone fruit orchards in Western Australia (Table S1). Conidia were collected in a drop of water, then spread on 1% water agar media to separate single spores using a microscope. Individual spores were transferred to V8 agar medium (V8 juice 200 mL, distilled water 800 mL, agar 15 g) and incubated in the dark at 22 °C. After a week, a 5 × 5 mm square of agar containing actively-growing hyphae was excised from the edge of the mycelium and transferred to V8 liquid medium, and placed on a shaker at 100 rpm in the dark at 22 °C. After about a week, DNA and RNA were extracted for further studies.

### 2.2. DNA and RNA Extraction

Nucleic acids were extracted from 100 mg of mycelium, which was frozen in liquid nitrogen and ground to a fine powder using a mortar and pestle. The powdered mycelium was transferred into a 1.5 mL centrifuge tube containing 450 µL extraction buffer (0.1 M NaCl, 50 mM Tris (hydroxymethyl) aminomethane (Tris) pH8.0, 0.5 mM Ethylenediaminetetraacetic acid (EDTA) pH8.0, 1% Sodium dodecyl sulfate (SDS), 1% Polyvinylpolypyrrolidone (PVPP) and 450 µL phenol-chloroform (50:50) saturated with Tris-EDTA buffer (pH 8.0). The mixture was homogenized before being centrifuged at room temperature for 2 min. After that, 400 µl of the aqueous phase was transferred to a new tube containing 400 µl of phenol-chloroform, then mixed and centrifuged for 2 min. Then, 300 µL of the aqueous phase containing nucleic acids was removed to a new tube containing 58 µL of absolute ethanol to which 200 mg of cellulose powder (CF11, Whatman) was added and mixed. After centrifuging at high speed for 1 min, the material was separated into two phases, the DNA-containing supernatant and the pellet containing RNA. The supernatant (250 µL) was transferred to a fresh tube and 20 µL 3M NaOAC pH5.2 and 625 µL absolute ethanol was added to precipitate DNA. The pellet containing RNA was washed three times with 750 µL application buffer (0.1 mM NaCl, 50 mM Tris pH8.0, 0.5 mM EDTA pH8.0, absolute ethanol). The RNA pellet was eluted in 450 µL elution buffer (0.1 mM NaCl, 50 mM Tris pH8.0, 0.5 mM EDTA pH8.0). The RNA solution (450 µL) was removed and precipitated after incubation at −20 °C in a new tube containing 1 mL absolute ethanol and 45 µL 3M NaOAC pH5.2 for RNA collection.

DNA was used to identify the fungal species based on the comparison of internal-transcribed spacer (ITS) region sequences of ribosomal RNA genes, as previously described [19]. RNA was used for virus identification in *M. fructicola* isolate M196 by high-throughput sequencing.

### 2.3. cDNA Synthesis, PCR Amplification and Library Preparation for High-Throughput Sequencing 

cDNA synthesis was carried out in 20 µL volume containing the following components: 4 µL 5× GoScript™ Buffer; 2 µL 0.1 mM DDT; 1 µL 10 mM deoxynucleotides; 1 µL GoScript™ reverse transcriptase (Promega Corporation, Sydney, Australia); 1 µL 10 mM Tris EDTA; 8 µL water; 1 µL RNA, and 1 µL random primer adaptor (5′-CGTACAGTTAGCAGGCNNNNNNNNNNNN-3′, where N is any nucleotide, annealed to the complement of the adaptor sequence added to cDNA molecules). The mixture was incubated at 25 °C for 10 min; 42 °C for 60 min; and 72 °C for 15 min. cDNA was amplified by PCR using primer 5′-CGTACAGTTAGCAGGC-3′, which annealed to the complement of the adaptor sequence added to cDNA molecules, in 20 µL volume of 10 µL 2× GoTaq® Green Master Mix (Promega Corporation, Sydney, Australia); 4 µL cDNA products; 5 µL water; 1 µL barcode primer. The reaction was carried out with an initial cycle of 5 min at 94 °C; 40 cycles of 10 min at 94 °C, 20 s at 45°C and 30 s at 72 °C, an extension cycle of 5 min at 72 °C, and a final cycle of 5 min at 37 °C. The library was purified, quantified, and paired-end sequenced on an Illumina MiSeq platform at the Australian Genome Research Facility.

### 2.4. Sequence Analysis

The sequences obtained were analyzed as previously described [21]. Briefly, in CLC Genomics Workbench (Qiagen, Sydney) the reads were trimmed using a quality score of 0.05 and of ambiguous bases, then of primer sequences and indices after assigning them to source bins. *De novo* assembly was carried out on the trimmed reads to form contigs >300 nt in length. Assembly parameters were word size of 40 and bubble size of 50. Contigs were compared to sequences lodged in GenBank (National Center for Biotechnology Information, NCBI) databases (https://blast.ncbi.nlm.nih.gov) using Blastn and Blastx [22] to identify virus-like sequences. Overlapping contigs were joined together when possible, and missing sequences were determined after designing specific primers to span the gaps (Table S2). Depth of sequencing was determined by mapping raw reads back to consensus sequences using the ‘map to reference’ function in Geneious v9.1.7 (Biomatters, Auckland, New Zealand) [23]. Annotation of the genomes was done manually in Geneious v9.1.7 after comparison with related sequences, and amino acid sequences were deduced from the nucleotide sequences of open reading frames (ORF).

Specific primers (Table S3) were designed from consensus sequences and used to confirm the presence of these three mycoviruses in other fungal isolates. When an amplicon was detected, both strands were sequenced using the Sanger method to confirm presence of the virus.

### 2.5. Generation of Isogenic Fungal Lines Free of Mycoviruses

Four methods were tested alone or in combination to eliminate viruses from fungal isolates:

(1). Cold treatment. Fungal mycelium was stored in 30% glycerol at −80 °C for two years before being recovered on V8 liquid medium.

(2). Temperature shock. Fungal mycelium was stored in 30% glycerol at −80 °C for two years was heated to 30 °C for 30 s, incubated in liquid nitrogen for 45 s, then heated again to 30 °C for 45 s. Mycelium was then recovered on V8 liquid medium.

(3). Hyphal tipping. Hyphal tips were harvested from the edges of rapidly-growing colonies, and sub-cultured to a fresh water agar plate.

(4). Antibiotic treatment. Antibiotics were added singly to water agar when the autoclaved media had cooled to approximately 50 °C. Antibiotic concentrations used were 12.5 mg/L cycloheximide, 100 mg/L kanamycin, and 250 mg/L streptomycin. Hyphal tips were inoculated to plates and cultures incubated in the dark at 22 °C for six days before hyphal tips were harvested from it and the process repeated. Each line was treated this way five times before it was tested for the presence of all three mycoviruses by RT-PCR using species-specific primers. Species-specific primers were designed from high-throughput sequences (Table S3).

Virus-free lines were maintained in culture for up to six months before their virus-free status was reconfirmed by RT-PCR with species-specific primers. These virus-free lines were used for subsequent growth rate and virulence experiments.

### 2.6. Virulence on Cherry

Isolate M196 (triple virus-infected) and isogenic M196 lines free of at least one virus (M196-1, M196-4, M196-6) (Table 1) were grown on V8 agar medium without antibiotics at 22 °C for six days before a 2 × 2 mm square of mycelium was harvested from the margin of the colony. This was placed on the surface of a washed and dry cherry fruit (*Prunus avium*) of cultivar Bing. Inoculated fruits were incubated in 100% humidity in the dark at room temperature (17–20 °C) for seven days. Each fungal isolate was inoculated to 36 cherry fruits; each fruit was treated as a replication. At the end of the incubation period, the widest extent of the fungal lesion was measured using a compass and ruler. The entire experiment was repeated twice.

### 2.7. Mycelial Growth In Vitro

Four virus-free and virus-infected isogenic lines (M196, M196-1, M196-4, M196-6) were grown on V8 agar plates at 22 °C for six days. Each isolate was inoculated into 36 V8 plates (three replications × 12 plates per replication). After six days, the maximum diameter of each colony was measured and differences in morphology recorded. The entire experiment was repeated twice.

To determine the differences between treatments, a one-way analysis of variance (ANOVA) was done at a significance level of 0.05 using SPSS version 24.

## 3. Results

### 3.1. Sequencing Analysis and Virus Assays

A MiSeq sequencing run created 13,264,058 reads of 100 nt. Barcode (index) sequences were used to assign reads to samples. After screening for quality and trimming the barcode and primer sequences, *de novo* assembly resulted in 37,146 contigs within the size ranges of 300 to 5385 nucleotides. Blastn and Blastx analysis of the contigs revealed 20 that shared nucleotide or amino acid identities with viral sequences. Where two or more contigs mapped to the same virus, gaps in the sequence were filled by RT-PCR so that the sequences of complete or almost complete genomes were determined (Table S2). Three previously-identified mycoviruses infected *M. fructicola* M196 (Table S1). RT-PCR assays using species-specific primers (Table S3) with appropriate controls were used to confirm the presence of the viruses and to check for their presence in isogenic lines over time. Isogenic lines of isolate M196 lacking one, two or three viruses were maintained in culture for 26 weeks before in vitro and *in planta* growth rates were measured.

#### 3.1.1. Sclerotinia sclerotiorum Hypovirus 2

A contiguous virus-like sequence of 13,535 nucleotides was obtained from *M. fructicola* isolate M196. The nucleotide and deduced amino acid sequences shared the greatest identities with the replicase gene of three previously described isolates of Sclerotinia sclerotiorum hypovirus 2 (SsHV2) (genus *Hypovirus*, family *Hypoviridae*) classified as a ssRNA virus. Alignment of the new sequence revealed that it shared 85–89% pairwise nucleotide and 93–95% amino acid identities with SsHV2 isolates 5427 from New Zealand (KF525367) [24], isolate SsHV2 from the USA (KF898354) [25], and isolate SX247 from China (KJ561218) [13]. The demarcation criterion for hypovirus species is less than 50% pairwise nucleotide identity over the complete genome [26]. Although identity with Rosellinia necatrix hypovirus 2 is 54%, identities with other SsHV2 isolates is far higher, and so we propose this to be a member of species *Sclerotinia sclerotiorum hypovirus 2.* It is designated Sclerotinia sclerotiorum hypovirus 2 isolate Monilinia-TNS. SsHV2 was identified only in *Monilinia* M196.

The sequence of SsHV2-Monilinia-TNS is estimated to represent 93% of the complete genome. The SsHV2-Monilinia-TNS genome comprises one large ORF, which is incomplete at the 5′end. The ORF extended from nucleotide 1–13,205 where it was terminated by an opal stop codon (UGA). The ORF is followed by a 3′ untranslated region (UTR) of 333 nucleotides, present from nucleotides 13,206 to 13,535. The conserved RdRp core motifs V and VI (S/TG x3 T x3 NS/T x22 GDD) (where x is any amino acid residue) [27] was present as TG x3 T x3 DS x38 GDD. No poly(A) tail region was detected. The SsHV-2 sequence was assigned GenBank accession MH665657.

#### 3.1.2. Fusarium poae Virus 1

Two contigs representing the complete genomic segments of a bipartite virus were identified from *M. fructicola* M196. The sequence shared the highest sequence identity with published isolates of Fusarium poae virus 1 (FpV1) (genus *Partitivirus*, family *Partitviridae*), a double-stranded RNA virus. One segment (RNA1) encodes the replicase (RdRp) and the other (RNA2) encodes the coat protein. Comparison of the 2100 nucleotide sequence of RNA1 revealed that it shared 90% pairwise identity with RNA1 of two FpV1 isolates: A11 from Slovakia (AF047013) and 240374 from Japan (LC150606). The deduced amino acid sequence of the RdRp segment shared 92% identity with the RdRps of these two isolates. The ORF of RNA1 extended from nucleotide 54-2084 where it was terminated by an opal stop codon, encoding an RdRp-like protein of 689 amino acid residues with an estimated molecular weight of 79.9 kDa. The conserved core RdRp motifs V and VI [28] were present as SG x3 T x3 DS x29 GDD. The 5′UTR extended from nucleotide 1 to 53 and the 3′UTR from nucleotide 2085 to 2160.

The RNA2 segment, encoding the coat protein (CP) gene, was 2093 nucleotides in length. The single ORF encoding a protein deduced to be 654 amino acid residues in length, with an estimated mass of 72.6 kDa. Surprisingly, a partial RdRp-like motif (T x2 DS x27 GDD) was present with the CP ORF. The deduced amino acid sequence of ORF1 of RNA2 shared greatest identity (85–86%) with CPs of the two other FpV1 isolates, and the nucleotide identity was 83–84% with them. The 5′ UTR extended from 1 to 102 nt, and the 3′ UTR from 2017 to 2093 nt.

The levels of identity of this new virus with isolates of FpV1 were above the species demarcation of partitiviruses recommended by the ICTV [29] of <40% amino acid identity between the RdRps, and so the isolate is proposed here as a member of species *Fusarium poae virus 1*. The isolate from M196 was designated Fusarium poae virus 1 isolate Monilinia-TNS. FpV1-Monilinia-TNS RNA1 was assigned GenBank accession MH665658, and FpV1-Monilinia-TNS RNA2 assigned GenBank accession MH665659.

#### 3.1.3. Botrytis Virus F

Contigs representing partial genomic sequences of a virus were detected from *M. fructicola* M196. The deduced amino acid sequences of fragments each shared 90–95% identity to those of an isolate of Botrytis virus F (BVF) (accession NP068550) (genus *Mycoflexivirus*, family *Gammaflexiviridae*), a ssRNA virus. Together, the three fragments were estimated to represent about 30% of the BVF complete genome, designated Botrytis virus F isolate Monilinia-TNS. One of the fragments held the core RdRp motifs V and VI as SG x3 T x3 NT x21 GDD. The three contigs of BVF-Monilinia-TNS were assigned GenBank accessions MH665660, MH665661, and MH665662.

### 3.2. The Presence of Mycoviruses in Other Monilinia Isolates

Eighteen *M. laxa* isolates and nine *M. fructicola* isolates were screened using SsHV2, FpV1 and BVF-specific primers (Table S3). Three *M. laxa* isolates harbored one or more of these viruses, including M82 (Fusarium poae virus 1), M84 (Fusarium poae virus 1 and Botrytis virus F), and M140 (Fusarium poae virus 1) (Table S1), but none of the other nine *M. fructicola* isolates tested held these viruses.

### 3.3. Elimination of Mycoviruses

The cold treatment and temperature shock methods failed to eliminate any mycoviruses, and the temperature shock method resulted in the death of most cultures (Table 1). On the other hand, hyphal tipping with and without antibiotics was effective at curing *M. fructicola* M196 of one or more of the three mycoviruses. It is unclear whether any of the antibiotics played a significant role in eliminating the viruses because tipping alone without antibiotics generated isogenic line M196-1 that was free of all three mycoviruses. Treatment with 12.5 mL/L cycloheximide eliminated SsHV2 from two isogenic lines, one of which, M196-4, was used in subsequent experiments. Treatment with kanamycin (100 mg/L), or with 250 mg/L streptomycin, eliminated both SsHV2 and BVF, but not the partitivirus FpV1 (Table 2). All isogenic lines were assayed by RT-PCR using virus-specific primers (Table S3, Figure S1) before and after subsequent experiments, and these tests confirmed the maintenance of their virus status.

### 3.4. Mycoviruses Influenced Growth In Vitro

Triple-infected parent isolate M196 grew significantly faster (*p* < 0.05) than lines lacking one or more viruses. After six days in culture, the mean diameter of colonies of isolate M196 was 73.8 mm, while virus-cured lines M196-1, M196-4, and M196-6 were less: 66.4, 62.9, and 66.0 mm, respectively (Table 2).

Where SsHV2 was absent but FpV1 and BVF were present (M196-4), colony growth was suppressed, suggesting that SsHV2 enhanced growth. There was no significant difference (P > 0.05) in growth between virus-free line M196-1 and FpV1-infected M196-6, indicating that FpV1 had no influence on growth. Where FpV1 and BVF co-occurred (M196-4), mycelial growth was suppressed compared to virus-free line M196-1. Although FpV1 alone had no apparent effect on growth, in combination with BVF it suppressed growth. This result could be interpreted as BVF alone suppressing growth and FpV1 remaining latent or as a synergistic suppressive influence of both viruses (Table 2, Table 3, and Table 4, Figure S2).

### 3.5. Influence of Mycoviruses on Virulence of M. fructicola

When cherry fruits were inoculated with the four isogenic lines of M196, there was no significant difference (*p* > 0.05) in the diameters of the resulting lesions. Although mean lesion size was lowest for M196 (8.7 mm), and virus-cured lines grew faster (9.6, 8.9, and 10.9 mm for M196-1, M196-4, and M196-6, respectively) (Table 3), they were not significantly different (*p* > 0.05) (Table 3). Mock inoculated fruits used as controls never became infected with *Monilinia* or other pathogens during the course of the experiment.

## 4. Discussion

The *M. fructicola* and *M. laxa* isolates tested were not widely infected with mycoviruses. Of the 18 *M. laxa* isolates and 10 *M. fructicola* isolates tested, only three *M. laxa* and one *M. fructicola* isolate were infected with one or more of three mycoviruses. This overall infection rate of 14% is far below that reported in the only other study of mycoviruses from *Monilinia* [20]. That study reported 76% of *M. fructicola* isolates tested were infected with at least one mycovirus. This report was based on RNA profiles and visualization of virus-like particles, not by sequence analysis [20]. Visualization of RNA species by electrophoresis or virus particles by TEM may not be sensitive enough to detect very low-titer viruses or those lacking a coat protein, and it provides only clues to identity based on genome size and/or virion shape and size. A shotgun sequencing approach, as was used here, should be capable of detecting all RNA-based viruses present, and the resulting sequence provide evidence for taxonomic placement.

The genome sequences of SsHV2 and FpV1 obtained using this approach were complete or almost complete, while that of BVF was partial. The incomplete genome may be a function of the low titer of BVF relative to the other two viruses, but it is possibly a function of differential RNA extraction efficiencies of the extraction procedure (cellulose-based) used.

*Monilinia fructicola* isolate M196 was infected with three mycoviruses, all of which were originally identified from other host genera, and none had previously been identified from Australia. Their presence in Australia probably reflects anthropogenic international translocations of mycovirus-infected *M. fructicola* isolates infecting *Prunus* fruit and germplasm, although the other known hosts of these mycoviruses, *Sclerotinia sclerotiorum, Fusarium poae* and *Botrytis cinerea*, all occur in Australia and may carry these viruses. Notably, *M. fructicola* and other known fungal hosts of the three viruses identified all have international distribution, they are all serious plant pathogens, and they are all members of the family Sclerotiniaceae [30].

Until now, SsHV2 has been identified in *S. sclerotiorum* in China, New Zealand and the USA [8,25], but not from *Monilinia* and not from Australia. Two groups have shown SsHV2 induces hypovirulence in its host. A study with isogenic lines of *S. sclerotiorum* infected with SsHV2 and an endornavirus showed that the presence of these mycoviruses reduced mycelial pigmentation and sclerotia formation in vitro. SsHV2 induced hypovirulence of *S. sclerotiorum* on lettuce and soybean, delaying production of sclerotia and reducing their numbers, but its influences on mycelial growth rate or virulence were not recorded [25]. Hu et al. [13] reported SsHV2 was associated with hypovirulence of *S. sclerotiorum* on canola (*Brassica napus*) in China. In contrast, our studies with SsHV2 infecting a *M. fructicola* line (co-infected with with FpV-1 and BVF) did not indicate hypovirulence. Our studies suggest SsHV2 enhanced *Monilinia* mycelial growth in vitro, it did not visibly change pigmentation or conidia production, and it did not appear to influence virulence on cherry. Unfortunately, we were unable to generate a line containing only SsHV2 to confirm these findings.

This is the first report of the betapartitvirus FpV1 being identified in a host beyond *Fusarium poae,* and its first report from Australia [29]. In *F. poae* [31] and here in *M. fructicola*, FpV1 does not seem to induce abnormal morphology or changes to virulence, and this is a common observance for partitiviruses generally [1]. Known exceptions are Aspergillus fumigatus partitivirus-1 (AfuPV-1) which induced abnormal aconidial sectors and a light pigmentation phenotype in *Aspergillus fumigatus* [28], and Sclerotinia sclerotiorum partitivirus-1 (SsPV1) that induced a hypovirulence phenotype in *Sclerotinia sclerotiorum* after damaging cell organelles [8].

*Botrytis virus F* is the sole species in genus *Mycoflexivirus*, family *Gammaflexiviridae*, a group closely aligned to other fungus-infecting viruses in the *Deltaflexiviridae* and to plant-infecting viruses in the *Alphaflexiviridae* and *Betaflexiviridae* [32]. BVF was the first virus identified from *Botrytis cinerea,* on strawberries in New Zealand [33]. It was also identified from a fungus associated with grapes in South Africa [34]. Our results suggest that BVF slightly reduced *M. fructicola* growth in vitro. That BVF may negatively impact growth of *M. fructicola* is of potential interest, given that both *B. cinerea* and *M. fructicola* are important plant pathogens, together affecting over 200 plant species [35,36]. The possibility that BVF induces hypovirulence should be studied further.

Investigation of the influence of mycoviruses on the growth of *M. fructicola* is not a trivial exercise, involving ‘curing’ parent fungal isolates of viruses to obtain isogenic lines [37]. Culturing fungi with cycloheximide is used widely to eliminate RNA mycoviruses [38]; however, this approach is not always successful. Incubation of *Aspergillus niger* cultures infected with a number of virus-like particles on cycloheximide failed to eliminate any of the viruses [39,40]. It was unclear if the antibiotics used in our experiments had any influence on virus elimination because hyphal tip culture alone without antibiotics effectively cured line M196-1 of all three viruses.

A limitation of our experiment is that conclusions of hypervirulence are based on linear colony growth measurements, not on measurements of total biomass, metabolism, or fecundity. It is unclear how the triple-infection by viruses might stimulate *M. fructicola* growth in vitro, but not affect virulence on fruit. In another study, growth of a chryso-like-virus-infected strain of *Alternaria alternate* in vitro was severely restricted, while virulence against fruit was enhanced [41]. The agar plate and the fruit are very different environments. On the plate, the complex cellular interactions between the fungal cells and plant cells is lacking [42,43,44]. We can only speculate that infection by one or more of these viruses, probably SsHV1, provided a means by which *M. fructicola* could metabolize nutrients more efficiently on the plate than could the virus-free isogenic line, but the mechanism for this is unknown.

## Figures and Tables

**Table 1:** Presence (+) or absence (−) of mycoviruses from isogenic lines of *M. fructicola* M196 after treatment. The antibiotic treatments were combined with hyphal tipping.

Mycoviruses ^a^	−80 °C Storage	Temperature Shock	Hyphal Tipping	Cycloheximide	Kanamycin	Streptomycin
SsHV2	+	+	−	−	−	−
FpV1	+	+	−	+	+	+
BVF	+	+	−	+	−	−
Number of lines obtained ^b^	6	1	1	2	1	1
Name of line	M196	−	M196-1	M196-4	M196-6	−

^a^ SsHV2, Sclerotinia sclerotiorum hypovirus 2; FpV1, Fusarium poae virus 1; BVF, Botrytis virus F. ^b^ Number of isogenic lines showing the given pattern out of six treated lines sub-cultured from parent isolate M196. ^c^ Name and source of line used in subsequent experiments.

**Table 2:** Summary of colony diameter on V8 medium and lesion diameter on cherry of isogenic *M. fructicola* lines.

			Colony Diameter on V8 Medium			Lesion Diameter on Cherry Fruit		
Line	Treatment ^a^	Viruses present ^b^	Range (mm)	Mean (mm)	Variance	Range (mm)	Mean (mm)	Variance
M196	None	SsHV2; FpV1; BVF	68–80	73.8	11.8	3–21	8.7	26.5
M196-1	Hyphal tipping	None	56–70	66.4	12	3–22	9.6	33.7
M196-4	Cycloheximide	FpV1; BVF	55–70	62.9	16.9	3–21	8.9	29.5
M196-6	Kanamycin	FpV1	60–71	66.0	11.5	3–25	10.9	33.4

^a^ Antibiotic treatments were combined with hyphal tipping; ^b^ SsHV2, Sclerotinia sclerotiorum hypovirus 2; FpV1, Fusarium poae virus 1; BVF, Botrytis virus F.

**Table 3:** *p*-values of paired comparisons between *M. fructicola* isogenic lines of colony diameter on V8 medium and lesion diameter on cherry fruits.

Line ^a^	V8 Medium			Cherry Fruit		
	M196	M196-1	M196-4	M196	M196-1	M196-4
M196-1	2.20e^−13^	-		0.5	-	
M196-4	4.30e^−19^	1.50e^−04^	-	0.8	0.6	-
M196-6	1.60e^−14^	0.6	0.0007	0.09	0.4	0.1

*p*-value < 0.05 indicates a significant difference between means. ^a^ Isolate M196 contains SsHV2-Monilinia-TNS, FpV1-Monilinia-TNS, BVF-Monilinia-TNS, isogenic M196-1 line is virus-free, M196-4 line contains FpV1-Monilinia-TNS, BVF-Monilinia-TNS, M196-6-Monilinia-TNS line carries FpV1-Monilinia-TNS.

**Table 4:** Possible effects on mycelial growth by each mycovirus.

Virus	V8 Medium	Cherry Fruit
SsHV2-Monilinia-TNS	Increase mycelial growth	No effect on lesion
FpV1-Monilinia-TNS	No effect on mycelial growth	No effect on lesion
BVF-Monilinia-TNS	Decreases mycelial growth	No effect on lesion

## Supplementary Materials

The following are available online at http://www.mdpi.com/1999-4915/11/1/89/s1, Figure S1: Presence and absence of mycoviruses in isogenic fungal lines treated to remove mycoviruses; Figure S2. Comparison of typical plates of four isogenic lines of *M. fructicola* isolate M196 inoculated on V8 media after 5 days incubation in the dark at 25 °C; Table S1. *Monilinia* isolates from Western Australia and their mycovirus-infection status; Table S2. Primers used to fill the gaps of the virus sequences; Table S3: Species-specfic primers used to reconfirm the presences of mycoviruses in fungal hosts.
