# Supplementary material for: Co-Infection with Three Mycoviruses Stimulates Growth of a Monilinia fructicola Isolate on Nutrient Medium, but Does Not Induce Hypervirulence in a Natural Host
Source: Viruses. 2019 Jan 21;11(1):89. doi: 10.3390/v11010089 (PMC6356717; doi:10.3390/v11010089)
Supplement: Supplementary file 1 [file viruses-11-00089-s001.pdf]

Supplementary Materials:

Table S1. *Monilinia* isolates from Western Australia and their mycovirus-infection status.

Isolate code	*Monilinia* species	Prunus host	Region of collection	Collection year	Mycoviruses detected[a]
M50	*M. laxa*	*P. domestica*	Perth Hills	2016	none
M52	*M. laxa*	*P. persica*	Perth Hills	2016	none
M53	*M. laxa*	*P. persica*	Perth Hills	2016	none
M61	*M. laxa*	*P. armeniaca*	Pemberton	2016	none
M67	*M. laxa*	*P. domestica*	Pemberton	2016	none
M68	*M. laxa*	*P. domestica*	Nannup	2016	none
M81	*M. laxa*	*P. persica*	Perth Hills	2016	none
M82	*M. laxa*	*P. persica*	Perth Hills	2016	FpV1
M84	*M. laxa*	*P. persica*	Perth Hills	2016	FpV1, BVF
M112	*M. laxa*	*P. armeniaca*	Perth Hills	2016	none
M123	*M. laxa*	*P. persica*	Perth Hills	2016	none
M128	*M. laxa*	*P. persica*	Pemberton	2016	none
M133	*M. laxa*	*P. persica*	Perth Hills	2016	none
M136	*M. laxa*	*P. persica*	Perth Hills	2016	none
M138	*M. laxa*	*P. armeniaca*	Donnybrook	2016	none
M139	*M. laxa*	*P. armeniaca*	Donnybrook	2016	none
M140	*M. laxa*	*P. armeniaca*	Donnybrook	2016	BVF
M141	*M. laxa*	*P. armeniaca*	Donnybrook	2016	none
M186	*M. fructicola*	*P. persica*	Perth Hills	2017	none
M187	*M. fructicola*	*P. persica*	Perth Hills	2017	none
M188	*M. fructicola*	*P. persica*	Perth Hills	2017	none
M189	*M. fructicola*	*P. armeniaca*	Perth Hills	2017	none
M191	*M. fructicola*	*P. armeniaca*	Perth Hills	2017	none
M192	*M. fructicola*	*P. armeniaca*	Perth Hills	2017	none
M193	*M. fructicola*	*P. armeniaca*	Perth Hills	2017	none
M194	*M. fructicola*	*P. armeniaca*	Perth Hills	2017	none
M195	*M. fructicola*	*P. persica*	Kirup	2017	none
M196	*M. fructicola*	*P. persica*	Kirup	2017	SsHV2, FpV1, BVF

a. SsHV2, Sclerotinia sclerotiorum hypovirus 2; FpV1, Fusarium poae virus 1; BVF, Botrytis virus F.

Table S2. Primers used to fill the gaps of the virus sequences.

Mycoviruses	Primer names	Primer sequences
Sclerotinia sclerotiorum hypovirus 2 (SsHV2-Monilinia-TNS)	M196HypoF1	ATGAAGAAGGATTTCCGTGA
	M196HypoF2	TTATCCAACTGTCCGGGCTC
	M196HypoR2	GAGCCCGGACAGTTGGATAA
	M196HypoF3	AATTGCGACCTTTCAGTTTC
	M196HypoR3	GAAACTGAAAGGTCGCAATT
	M196HypoF4	TACGCAAGAGCATCCGGTTC
	M196HypoR4	GAACCGGATGCTCTTGCGTA
	M196HypoF5	TGACTCGGCAGAAGAATTGT
	M196HypoR5	ACAATTCTTCTGCCGAGTCA
	M196HypoF6	GAAAGCTTCGCAACAAGAAG
	M196HypoR6	CTTCTTGTTGCGAAGCTTTC

		M196HypoF7	TATGATCCGCAGATACATGT
		M196HypoR7	ACATGTATCTGCGGATCATA
		M196HypoF8	TATTTAAGGAAATGGGACCG
		M196HypoR8	CGGTCCCATTTCCTTAAATA
		M196HypoF9	ATGTGGCCTATCCCTTCCAA
		M196HypoR9	TTGGAAGGGATAGGCCACAT
		M196HypoF10	GCCAAGAATGAATGGATATG
		M196HypoR10	CATATCCATTCATTCTTGGC
		M196HypoF11	CATATCCATTCATTCTTGGC
		M196HypoR11	GCCAAGAATGAATGGATATG
		M196HypoF12	GAGGTGACAGGGTGCCTGTT
		M196HypoF12	AACAGGCACCCTGTCACCTC
		M196HypoF13	AGTATAAAAAGGTGTACACC
		M196HypoR13	GGTGTACACCTTTTTATACT
		M196HypoF14	CAATATGTTTATCTTTTGGC
		M196HypoR14	GCCAAAAGATAAACATATTG
		M196HypoF15	GATGACCTTCCAGATGAAGA
		M196HypoR15	TCTTCATCTGGAAGGTCATC
		M196HypoF16	AATGAAGCCTTCGAAGAAGG
		M196HypoR16	CCTTCTTCGAAGGCTTCATT
		M196HypoF17	TCTTGAACGGGAGCATTTCT
		M196HypoR17	AGAAATGCTCCCGTTCAAGA
		M196HypoF18	ATGGGAATGTTTACAAGGAT
		M196HypoR18	ATCCTTGTAAACATTCCCAT
		M196HypoF19	TTGAGCGTGTGGCAAGGCCT
		M196HypoR19	AGGCCTTGCCACACGCTCAA
		M196HypoF20	GATGCCCATCGCATGATTGA
		M196HypoR20	TCAATCATGCGATGGGCATC
		M196HypoF21	AGACAGGATGCATTCGTCAA
		M196HypoR21	TTGACGAATGCATCCTGTCT
		M196HypoF22	TAATCTGGAACACGCAACGA
		M196HypoR22	TCGTTGCGTGTTCCAGATTA
		M196HypoR23	TCATCTGTGGCGTTTCTCCT
		M196HypoR24	ATTTCCTGCAATACGTAGTC
		Gaphypo5	ATCATACGGAGTGCCTCACT
		Gaphypo3	AGGGTGACTCTAGATACACA
		5'hypo2	CCTGTTGTTACCTGTCAGAC
		5'hypo1	AGTAGTGTCCTAGAAGAATG
		3'hypoR2	CTTCGTCAAGATTGTCGATC
		3'hypoR1	AGAACGCGTCACGTTCCCGC
	Botrytis virus F (BVF-Monilinia-TNS)	M196BotVF5	AAACGCCUGAAUCGUACGGCCACC
		M196BotVF800R	TTGGATATGACAAAGATATGG
		M196BotVF800F	CCATATCTTTGTCATATCCAA
		M196BotVF1500F	CTTGCCACTATCACTGCCGC
		M196BotVF1500R	AGTCGAGCATATTGCTTAGC
		M196BotVF3000F	GCTAAGCAATATGCTCGACT
		M196BotVF2200R	GCGTAGACTTCTTCAGTGAG
		M196BotVF2200F	CTCACTGAAGAAGTCTACGC
		M196BotVF1500R	GCGGCAGTGATAGTGGCAAG
		M196BotV6800R	CCTCGTGTGCAACGAAGTGT
		M196BotV6300R	TCATTGAAGTCGATGCACAC
		M196BotV6300F	GTGTGCATCGACTTCAATGA
		M196BotV5500R	GGCGTACCGTGTGCGCCAGA
		M196BotV5500F	TCTGGCGCACACGGTACGCC

M196BotV4700R	CAGTGCGGCATTCTTCCAGC
M196BotV4700F	GCTGGAAGAATGCCGCACTG
M196BotV3800R	CGCGTATGTGTAAACCTTGC
M196BotV3800F	GCAAGGTTTACACATACGCG

Table S3: Species-specfic primers used to reconfirm the presences of mycoviruses in fungal hosts.

**Mycoviruses**	**Genes**	**Primer names**	**Primer sequences**
Sclerotinia sclerotiorum hypovirus 2 (SsHV2-Monilinia-TNS)	RdRp	SsHV1F	ACAGAAGCATGGTCGCAAAG
	RdRp	SsHV1R	CACGAAGGTCAACGCTTCAA
Fusarium poae virus 1 (FpV1-Monilinia-TNS)	RdRp	FpV2F	CGCTCTTCCGTTATATCGCG
	RdRp	FPV2R	ATAACGTGTTGGATGCGGTG
	CP	FPV1F	ACATCGAACTTGACTCCGGT
	CP	FPV1R	TTGGTGCGCAGAGTAGAAGA
Botrytis virus F (BVF-Monilinia-TNS)	RdRp	BV1F	TCCCCTATCACCATACGCAC
	RdRp	BV1R	GCTAGGTAGTCTGCGGCTTA
	CP	BV2F	ACATAGCGAGCGCTTTCATT
	CP	BV2R	ATTCCGAAACGTGACTGGAG

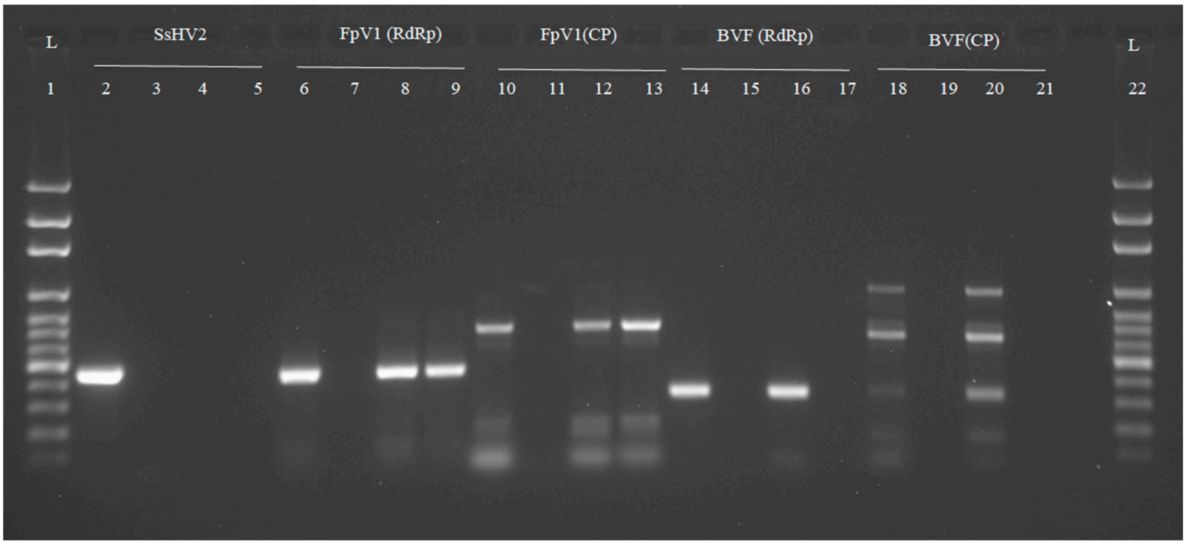

Figure S1. Presence and absence of mycoviruses in isogenic fungal lines treated to remove mycoviruses. Lanes 1 and 22: 100 bp DNA ladder. Five RT-PCR assays were done, each to test four isogenic lines, *viz* M196 (three viruses present), M196-1 (viruses absent), M196-4 (FpV1 and BVF present), and M196-6 (FpV1 present). Primers used are listed in Table S3. SsHV2 primers (lanes 2, 3, 4, 5); FpV1 (RdRp) primers (lanes 6, 7, 8, 9), FpV1 (CP) primers (lanes 10, 11, 12, 13), BVF (RdRp) primers (lanes 14, 15, 16, 17), BVF (CP) primers (lanes 18, 19, 20, 21).

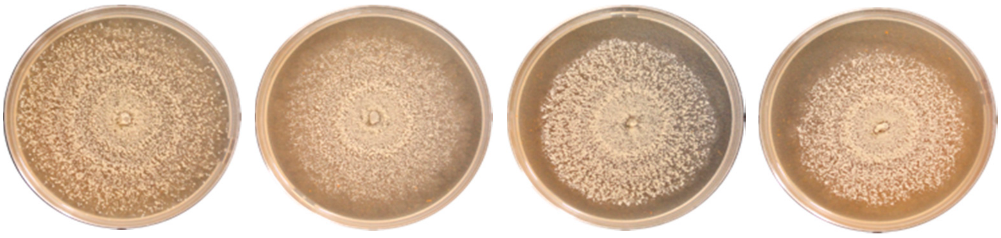

Figure S2. Comparison of typical plates of four isogenic lines of *M. fructicola* isolate M196 inoculated on V8 media after 5 days incubation in the dark at 25°C. Line M196 contains all three viruses, M196-1 contains no mycoviruses. M196-4 contains FpV1 and BVF and lacks SsHV2. M196-6 contains FpV1 and lacks SsHV2 and BVF.
